# Optimization of melanin production by *Brevundimonas* sp. SGJ using response surface methodology

**DOI:** 10.1007/s13205-012-0082-4

**Published:** 2012-08-07

**Authors:** Shripad N. Surwase, Shekhar B. Jadhav, Swapnil S. Phugare, Jyoti P. Jadhav

**Affiliations:** 1 Department of Microbiology, Shivaji University, Kolhapur, 416004 India; 2 Department of Biochemistry, Shivaji University, Kolhapur, 416004 India; 3 Department of Biotechnology, Shivaji University, Vidyanagar Kolhapur, 416004 India

**Keywords:** Melanin, Response surface methodology (RSM), *Brevundimonas* sp. SGJ, l-Tyrosine, EPR

## Abstract

Melanins are predominantly indolic polymers which are extensively synthesized in animals, plants and microorganisms. It has wide applications in cosmetics, agriculture and medicine. In the present study, optimization of process parameters influencing melanin production was attempted using the response surface methodology (RSM) from *Brevundimonas* sp. SGJ. A Plackett–Burman design was used for screening of critical components, while further optimization was carried out using the Box–Behnken design. The optimum conditions observed were pH 5.31, tryptone 1.440 g l^−1^, l-tyrosine 1.872 g l^−1^ and CuSO_4_ 0.0366 g l^−1^. Statistical analysis revealed that the model is significant with model *F* value 29.03 and *R*^2^ value 0.9667. The optimization of process parameters using RSM resulted in a 3.05-fold increase in the yield of melanin. The intermittent addition of l-tyrosine enhanced the melanin yield to 6.811 g l^−1^. The highest tyrosinase activity observed was 2,471 U mg^−1^ at the 18th hour of the incubation period with dry cell weight of 0.711 g l^−1^. The melanin production was confirmed by UV–Visible spectroscopy, FTIR and EPR analysis. Thus, *Brevundimonas* sp. SGJ has the potential to be a new source for the production of melanin.

**Electronic supplementary material:**

The online version of this article (doi:10.1007/s13205-012-0082-4) contains supplementary material, which is available to authorized users.

## Introduction

Melanins are indolic polymers that are widely distributed throughout the animal and plant kingdoms and are also synthesized by microorganisms (Riley 1997; Plonka and Grabacka 2006). Melanin synthesis has been reported by various bacteria and fungi, including *Escherichia coli* W3110 (Lagunas-Muñoz et al. 2006), *Bacillus cereus* (Zhang et al. 2007) and *Klebsiella* sp. GSK (Shrishailnath et al. 2010). Fungal species that synthesize melanin include *Cryptococcus neoformans*, *Aspergillus fumigatus* and *Pneumocystis carinii* (Plonka and Grabacka 2006).

The synthesis of melanin in microorganisms has several functions besides UV protection. Melanin synthesis has been associated with virulence for a variety of pathogenic microbes such as *Cryptococcus neoformans* and *Burkholderia cepacia*. Melanin binds with the antibiotics and confers the emerging resistance of pathogenic bacteria to the antibiotics. Consequently, melanin and melanin synthesis pathways are potential targets for antimicrobial drug discovery (Plonka and Grabacka 2006; Nosanchuk and Casadevall 2006).

Melanins confer resistance to UV light by absorbing a broad range of the electromagnetic spectrum and preventing photo-induced damage. Consequently, melanins are widely used in cosmetics, photo protective creams, eyeglasses and protective agents in *Bacillus thuringenesis* insecticidal crystals (Zhang et al. 2007). The melanin producing microorganism can also be used for immobilization of radioactive waste such as uranium (Turick et al. 2008). In addition, melanin synthesis genes from bacteria have been used as reporter genes to screen recombinant bacterial strains (Adham et al. 2003). Other reports have showed the anti-HIV properties of melanin as well as their usefulness for photo voltage generation and fluorescence studies (Montefiori and Zhou 1991; Hanyz and Wróbel 2003). Melanoma poses an increasing health problem that affects about 40,000 patients each year in the United States and an estimated 100,000 worldwide. Earlier experiments in mice have concluded that fungal (*Cryptococcus neoformans*) melanin can be used to generate monoclonal antibodies (mAb) for the treatment of human metastatic melanoma. This antibody also binds human melanin since both fungal and human melanins have structural similarities (Dadachova et al. 2008).

The optimization of fermentation conditions, particularly physical and nutritional parameters, are of primary importance in the development of any fermentation process owing to their impact on the economy and practicability of the process. Medium optimization and physical conditions have been traditionally performed using one-factor-at-a-time method. The disadvantages of such a classical method are that it is time consuming, laborious and expensive; in addition, it fails to determine the combined effect of different factors. Hence, researchers are encouraged to apply statistical experimental approaches such as response surface methodology (RSM), which provide a great amount of information based on only a small number of experiments (Aghaie-Khouzania et al. 2012).

Given the potential uses of melanin and their high demand, the need for the statistical development of low cost process exists. The objective of this study was to screen and optimize the process parameters using Plackett–Burman design and Box–Behnken design of RSM in order to increase the production of melanin by the *Brevundimonas* sp. SGJ.

## Methods

### Chemicals and microorganism

Melanin (synthetic) and l-tyrosine were purchased from Sigma-Aldrich Co. (St. Louis, MO, USA) whereas other chemicals were procured from HiMedia (India). The melanin-producing bacterial strain was isolated from garden soil of Shivaji University, Kolhapur, India, using serial dilution technique on a nutrient agar (HiMedia, India) supplemented with 1 g l^−1^l-tyrosine.

### Melanin production

The nutrient broth (HiMedia, India) used for the cultivation of the isolated bacterium consisted of 5 g l^−1^ peptone, 1.5 g l^−1^ beef extract, 1.5 g l^−1^ yeast extract, and 0.5 g l^−1^ NaCl, supplemented with 1 g l^−1^l-tyrosine and with a pH of 7. The 6-h grown, 2-ml cell suspension was inoculated in 100 ml of the same medium for melanin production in 250-ml Erlenmeyer flasks. The flasks were kept in an incubator shaker at 30 °C and 120 rpm; melanin was assayed after 48 h. Melanin production in the broth was assayed spectrophotometrically at 475 nm using a calibration curve of standard synthetic melanin (Sigma-Aldrich, St. Louis, USA) (Hoti and Balaraman 1993).

### Experimental design and statistical analysis

#### Plackett–Burman design

A Plackett–Burman design was used to select the most critical media components for melanin production by *Brevundimonas* sp. SGJ. The factors affecting the yield of melanin were selected by screening various carbon sources, nitrogen sources, and mineral salts and physical conditions such as pH and temperature. In addition, some of these variables were selected from the primary literature review (Lagunas-Muñoz et al. 2006; Shrishailnath et al. 2010).

A total of 11 process parameters, including pH (*X*_1_), temperature (*X*_2_), tryptone (*X*_3_), yeast extract (*X*_4_), beef extract (*X*_5_), glucose (*X*_6_), l-tyrosine (*X*_7_), CuSO_4_ (*X*_8_), MgSO_4_ (*X*_9_), K_2_HPO_4_ (*X*_10_), and NaCl (*X*_11_) were added at two levels: low (−1) and high (+1). The low and high levels of these factors were taken as pH (5 and 7), temperature (20 and 40 °C), while levels of media components were tryptone (0.5 and 2.5 g l^−1^), yeast extract (0.5 and 2.5 g l^−1^), beef extract (0.5 and 2.5 g l^−1^), glucose (0.5 and 2.5 g l^−1^), l-tyrosine (0.5 and 2.5 g l^−1^), CuSO_4_ (0.01 and 0.05 g l^−1^), MgSO_4_ (0.001 and 0.005 g l^−1^), K_2_HPO_4_ (0.5 and 2.5 g l^−1^) and NaCl (0.1 and 0.5 g l^−1^). This design characterizes a model that identifies the significant variables when no interaction among the factors is expected (Plackett and Burman 1946; Wang and Lu 2005; Anderson and Whitcomb 2005). The design matrix created using the Design Expert software (version 8.0, Stat-Ease Inc., Minneapolis, USA) is presented in Table S1. Three replicates at the center point were also performed to find the curvature that may exist in the model and the pure experimental error, which shows lack-of-fit. The statistical significance of the first-order model was identified using Fisher’s test for analysis of variance (ANOVA) (Wang and Lu 2005).

#### Box–Behnken design

Once the critical factors were identified via screening, a Box–Behnken design for independent variables was used for further optimization. Four variables at three levels were used to fit a polynomial model (Box and Behnken 1960; Anderson and Whitcomb 2005; Wang and Lu 2005). A second-order model is designed such that the variance of *Y* is constant for all points equidistant from the center of the design. The Design Expert software (version 8.0, Stat-Ease Inc., Minneapolis, USA) was used in the experimental design and data analysis. Response surface graphs were obtained to understand the effect of the variables, individually and in combination, and to determine their optimum levels for maximum melanin production. All trials were performed in triplicate, and the average melanin yield was used as response *Y*.

#### Tyrosinase activity, biomass trend and melanin production

After validation of the experiment using the optimum process parameters generated by the Design Expert software (Stat-Ease Inc., USA), the melanin production was observed with the process parameters before optimization (nutrient broth with 1 g l^−1^l-tyrosine) and after optimization. The biomass trend, tyrosinase activity, and melanin production were observed at 6-h time intervals for up to 48 h. The tyrosinase activity was determined by the previously described method (Kandaswami and Vaidyanathan 1973; Ali et al. 2007). The final assay concentration in the 3.0 ml reaction mixture contained 50 mM potassium phosphate (pH 7.4), 0.17 mM catechol, 0.070 mM and l-ascorbic acid equilibrated to 25 °C. The Δ*A*_265_ nm was monitored until constant, and then 0.1 ml of the cell-free broth was added. The decrease in the Δ*A*_265_ nm was recorded for 1 min. The Δ*A*_265_ nm was obtained using the maximum linear rate for both the test and the control. One unit of tyrosinase activity was equal to a Δ*A*_265_ nm of 0.001 per min at pH 7.4 at 25 °C in a 3.0-ml reaction mixture containing l-catechol and l-ascorbic acid. The protein content in the cell-free broth was determined using Lowry et al’s (1951) method. The intermittent addition of optimal l-tyrosine concentration was carried at 6-h time interval from 24 to 36 h, rather than only an initial concentration (Lagunas-Muñoz et al. 2006).

#### Purification and analysis of melanin

Melanin was purified from cell-free extract by previously described methods (Zhang et al. 2007). The chemical characterization of purified pigment was carried out using tests described by earlier reports (Zhang et al. 2007; Shrishailnath et al. 2010). In order to confirm the purified pigment as melanin, the FTIR (Fourier transform infrared) analysis was carried out using FTIR spectrometer (Shimadzu, Japan) and the EPR analysis was carried out using method described earlier (Shrishailnath et al. 2010), at the Department of Biophysics & Center for Imaging Research, Medical College of Wisconsin, Milwaukee, WI, USA.

#### Results and discussion

Bacteria have been exploited as a major source of melanin with potential commercial applications in the fields of cosmetics, pharmaceuticals and agriculture. However, the detailed optimization of the process parameters for the melanin production has not been reported. These factors may play a vital role in the cost effectiveness of melanin production, so they were optimized in the current study. The isolated strain was identified as a novel bacterial species *Brevundimonas* sp. SGJ (NCBI Genbank accession no. HM998899). The phylogenic tree was constructed with MEGA4 software (AZ, USA) (Tamura et al. 2007; Surwase and Jadhav 2011) (Fig. S1a). The melanin-producing colonies of *Brevundimonas* sp. SGJ are shown in Fig. S1b while melanin production in optimized conditions is shown in Fig. S1c.

#### Plackett–Burman design for screening of critical factors

Statistical analysis using a Plackett–Burman design indicated that pH (*X*_1_), tryptone (*X*_3_), l-tyrosine (*X*_7_), and CuSO_4_ (*X*_8_) significantly affected the melanin production, with *p* values less than the significance level of 0.05. The remaining components were found to be insignificant, with *p* values above 0.05. The ‘Pareto chart’ (Fig. S2) showed that the value of l-tyrosine (*X*_7_) was above the ‘Bonferroni Limit’; this indicates it is certainly significant. Also the values of pH (*X*_1_), tryptone (*X*_3_) and CuSO_4_ (*X*_8_) were above the *t*-value limit that implies that these factors are possibly significant whereas the remaining factors were below the *t*-value limit which indicates their insignificance (Anderson and Whitcomb 2005). Experimental runs and their respective melanin yields are presented in Table S1. Statistical analysis of the responses was performed, as shown in Table S2. The model *F* value of 29.73 implies that the model is significant; there was only a 0.01 % chance that a model *F* value this large could occur due to noise. The “Adeq Precision” ratio of 4.303 obtained in this study indicates an adequate signal. Thus, this model can be used to navigate the design space. The *R*^2^ value observed for this model was 0.4549 and the “Pred *R*-Squared” of 0.2468 is in reasonable agreement with the “Adj *R*-Squared” of 0.1823. Regression analysis was done on the results, and a first-order polynomial equation was derived, representing melanin production as a function of the independent variables:1

Statistical analysis showed that it is not possible to evaluate the relationship between significant independent variables and the response by a first-order equation. Thus, the first-order model is not appropriate to predict the response. Indeed, further investigation could be conducted through a second-order model.

#### Optimization by response surface methodology

Optimization of process parameters was carried out using the Box–Behnken design with the parameters found to be significant from the Plackett–Burman design, including pH (*X*_1_), tryptone (*X*_3_), l-tyrosine (*X*_7_), and CuSO_4_ (*X*_8_). Table S3 presents the design matrix and the results of the 29 experiments carried out using the Box–Behnken design. The results obtained were submitted to ANOVA using the Design Expert software (version 8.0, Stat-Ease Inc., Minneapolis, USA), and the regression model was given as:2where *X*_1_ is pH, *X*_3_ is tryptone, *X*_7_ is l-tyrosine, and X_8_ is CuSO_4_. The ANOVA of the quadratic regression model (Table S3) demonstrated that Eq. 2 is a highly significant model (*p* = <0.005). The model *F* value of 29.03 implies that the model is significant. The goodness of fit of the model was checked using the determination coefficient (*R*^2^). In this case, the value of the *R*^2^ was 0.9667. The value of the adjusted *R*^2^ was 0.9334. It was in reasonable agreement with the predicted *R*^2^ (0.8212). The lack-of-fit value for regression Eq. 2 was not significant (0.1010), indicating that the model equation was adequate for predicting the melanin production under any combination of values of the variables. “Adeq Precision” measures the signal-to-noise ratio, with a ratio greater than four considered as desirable (Anderson and Whitcomb 2005). The “Adeq Precision” ratio of 15.958 obtained in this study indicates an adequate signal. Thus, this model can be used to navigate the design space (Table S4).

#### Interaction effects of variables

Statistical analysis using a Plackett–Burman design showed critical parameters affecting melanin yield and these parameters were further optimized by Box–Behnken design. The ANOVA showed the model is significant with significant statistical values. The graphical representation provides a method to visualize the relationship between the response and experimental levels of each variable and the type of interactions between test variables in order to deduce the optimum conditions (Wang and Lu 2005).

**Fig. 1:**
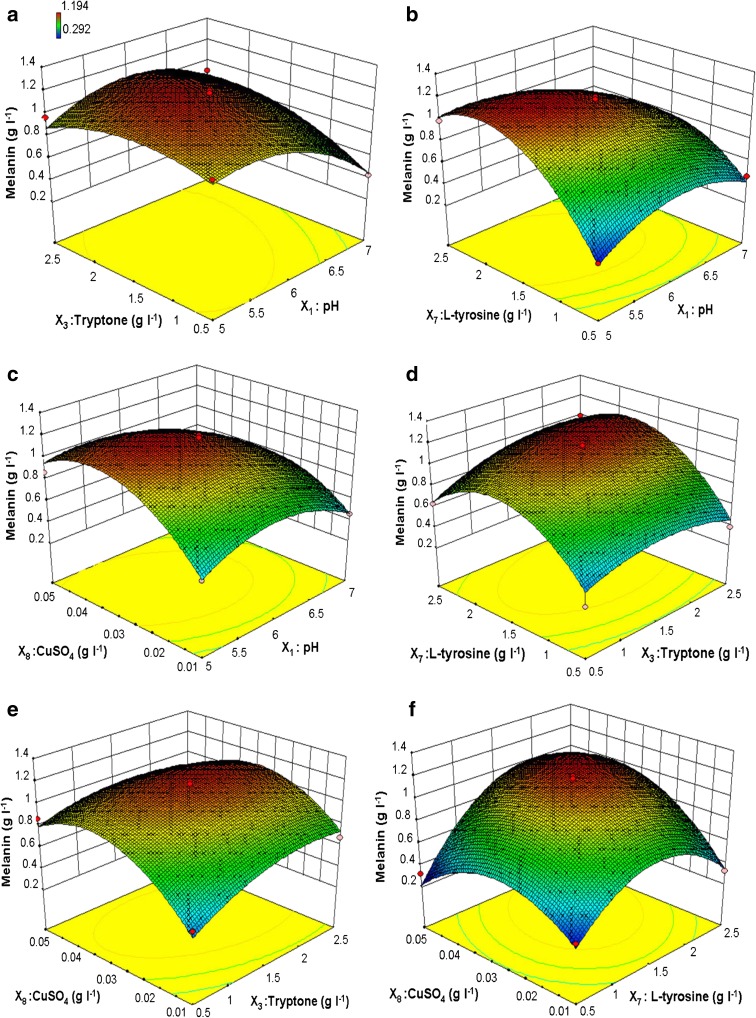
Three-dimensional response surface curve showing the effect of interactions of **a** pH and tryptone, **b** pH and l-tyrosine, **c** pH and CuSO_4_, **d** tryptone and l-tyrosine, **e** tryptone and CuSO_4_, **f**l-tyrosine and CuSO_4_

The interaction effects and optimal levels of the variables were determined by plotting the three-dimensional (3D) response surface curves. The response surface curve in Fig. 1a represents the interaction between pH and tryptone, which showed that the maximum melanin yield was obtained toward acidic pH while melanin yield was drastically affected with neutral pH. In addition, lower and higher levels of tryptone resulted in low yields of melanin. The optimum pH value obtained was slightly acidic while medium concentrations of the tryptone resulted in maximum yield. The shape of the response surface curves showed strong positive interaction between these tested variables. The interaction among pH and tryptone was significant, because slightly acidic pH in the present study might have enhanced the digestion of tryptone, which released peptides, leading to higher melanin yields. The pH optima for melanin production from earlier sources were in the range of 7–7.5. The optimum pH 7.5 have been reported for melanin production by *E. coli* (Lagunas-Muñoz et al. 2006) while pH 7.2 required by *Klebsiella* sp. GSK (Shrishailnath et al. 2010). In previous reports of melanin production, various organic nitrogen sources have been used in the medium, including casein for *Bacillus thuringiensis* (Chen et al. 2004), wheat flour and yeast flour, whereas bactotyptone and casein were used with *Bacillus cereus* (Zhang et al. 2007).

Figure 1b depicts the interaction of pH and l-tyrosine where the shape of the response surface curve indicates that melanin yield was mainly affected by interaction between these two factors. The slight alteration in concentration of these components leads to higher difference in the levels of produced melanin. Melanin production increased with acidic pH and higher l-tyrosine concentrations. The interaction between pH and l-tyrosine was found to be highly significant because l-tyrosine is the substrate for melanin production and its solubility decreases at neutral and alkaline conditions, while l-tyrosine is soluble at acidic conditions (Surwase and Jadhav 2011; Lagunas-Muñoz et al. 2006). The response surface curve for the interaction of pH and CuSO_4_ is represented in Fig. 1c. The shape of response surface curve shows a less significant interaction between these two variables. The melanin yield was found to be increased with higher concentrations of CuSO_4_ with slightly acidic pH. The results showed that melanin production was most affected by CuSO_4_ levels than pH. The pH and CuSO_4_ interaction showed that melanin production was more affected by CuSO_4_ because tyrosinase is a copper-containing enzyme (Claus and Decker 2006). Hence, the presence of CuSO_4_ in the medium increased the production of melanin by enhancing the enzymatic activity whereas higher concentration of CuSO_4_ might be toxic to the cells, which ultimately leads to decreased melanin yield.

The response surface curve of tryptone and l-tyrosine indicates that melanin yield was affected by the lower and higher levels of l-tyrosine while melanin production was less affected by tryptone levels (Fig. 1d). Thus 3D response curve as well as statistical analysis indicates the insignificant interaction between tryptone and l-tyrosine. Whereas less significant interaction was observed between tryptone and CuSO_4_ (Fig. 1d and e). The l-tyrosine and CuSO_4_ interaction (Fig. 1f) resulted in positive effect on melanin yield because l-tyrosine acts as substrate while CuSO_4_ acts as an inducer for the melanin synthesis pathway (Plonka and Grabacka 2006).

#### Validation of the model

Validation was carried out under conditions predicted by the model. The optimum conditions were pH 5.31, tryptone 1.440 g l^−1^, l-tyrosine 1.872 g l^−1^ and CuSO_4_ 0.0366 g l^−1^. The predicted yield of melanin with these values is 1.238 g l^−1^ and the actual yield obtained was 1.227 g l^−1^. The close correlation was seen between the experimental and predicted values which validate the model. The predicted yield of melanin by optimal levels of the variable generated by the model was in close correlation with experimental value, which signifies the RSM methodology over traditional optimization approach. In addition, the increased melanin production was observed with the parameters optimized using RSM than the initially used conditions.

#### Melanin yield, biomass trend and tyrosinase activity

**Fig. 2:**
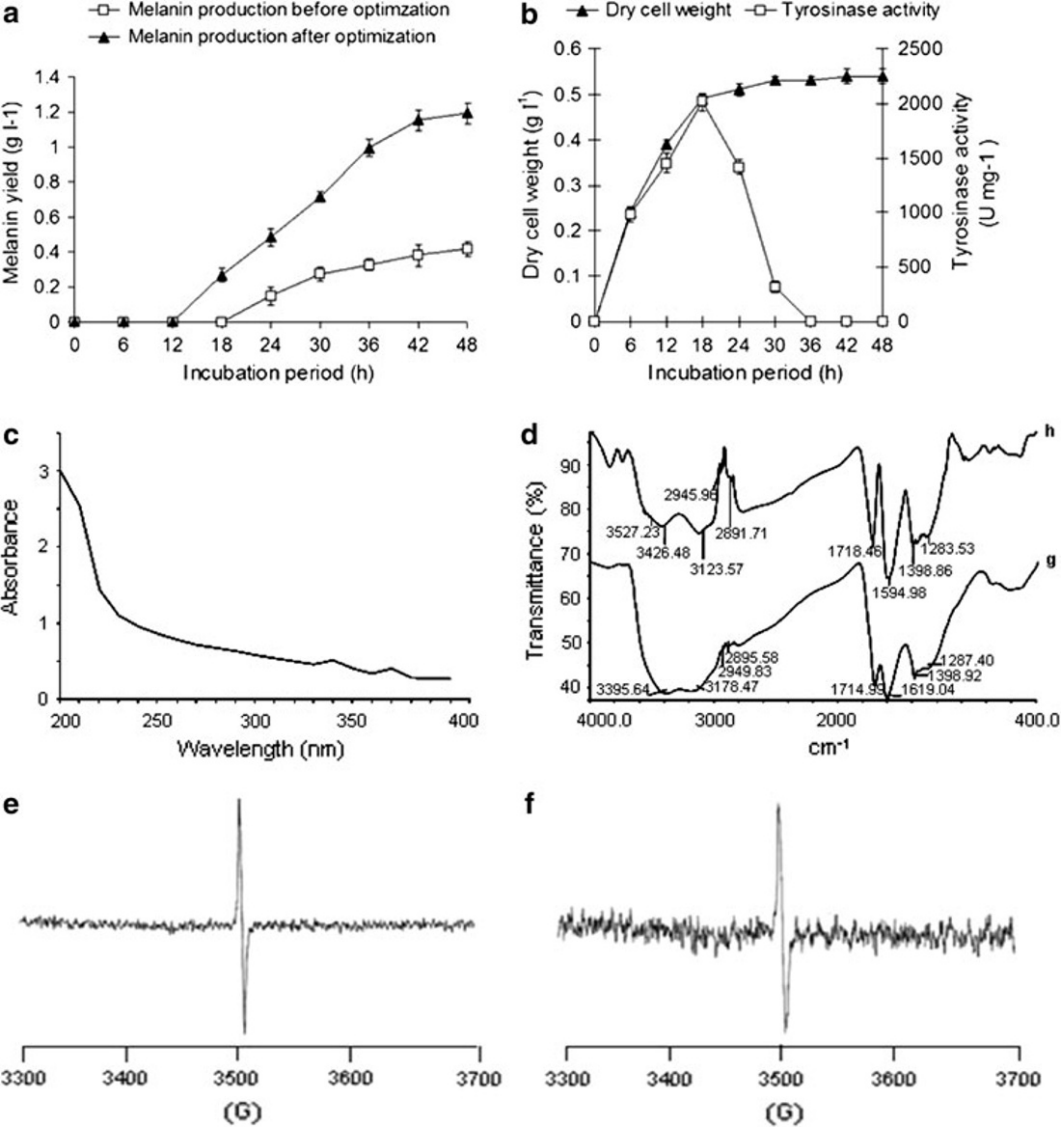
**a** Melanin production before and after optimization by RSM, **b** Dry cell weight and tyrosinase activity during melanin production, **c** UV- Visible spectroscopic analysis of bacterial melanin, **d** FTIR spectrum (*lower*) standard melanin (Sigma); (*upper*) bacterial melanin, **e** EPR spectrum of standard melanin (Sigma), **f** EPR spectrum of bacterial melanin

Melanin production before and after optimization is illustrated in Fig. 2a, which indicates that with process parameters before optimization, melanin production reached to 0.401 g l^−1^ at 48 h. The process parameters after optimization showed that melanin production gradually increased upto 1.227 g l^−1^ at 48 h. Thus, process optimization by RSM resulted in 3.05-fold increase in the melanin yield than the yield before optimization. The biomass trend and tyrosinase activity during melanin production with optimized parameters are depicted in Fig. 2b, which showed that dry cell weight increased gradually upto 24 h (0.521 g l^−1^) and after that, remains nearly constant with final weight of 0.540 g l^−1^, while tyrosinase activity increased slowly upto 2,015 U mg^−1^ at 18th hour and then decreased suddenly to 1,416 U mg^−1^ at 24th hour. The biomass trend during melanin production indicated that melanin production occurs in the stationary growth phase of bacterial cells. The decreased tyrosinase activity after 24 h might be due to conversion of l-tyrosine to l-DOPA and l-DOPA to dopaquinone, and substrates for the tyrosinase were not available further, during melanin synthesis pathway (Plonka and Grabacka 2006; Surwase and Jadhav 2011).

To enhance the yield of melanin, the intermittent addition of l-tyrosine was carried in optimized conditions as described previously (Lagunas-Muñoz et al. 2006). The 1.872 g l^−1^l-tyrosine was added intermittently with 6-h time interval from 24 to 36 h, which resulted in the highest yield of yield of 6.811 g l^−1^ melanin after 54 h. A literature survey revealed that *Brevundimonas* sp. SGJ used in this study produced the highest amount of melanin within the shortest incubation period (54 h). The other studies have reported isolation and characterization of melanin includes: *Klebsiella* sp. GSK which produced 0.540 g l^−1^ within 84 h of incubation, while melanin synthesis from Frankia strain Cel5 was about 0.180 g l^−1^ (Shrishailnath et al. 2010). On fungal strains, previous work was focused on the role of melanin synthesis in pathogenesis and virulence (Plonka and Grabacka 2006). As compared to earlier reports *Brevundimonas* sp. SGJ has several advantages over previously studied bacteria and fungi such as a shorter incubation period, efficient melanin production and simple and minimum medium components rather than complex medium requirements.

#### Analysis of melanin

The chemical characterization results showed that the purified dark brown powder was insoluble in water, 5 M HCl, ethanol, benzene, chloroform and acetone, while it was soluble in 1 M KOH and 1 M NaOH. It was decolorized after addition of H_2_O_2_; after the addition of KMnO_4_, the color of the pigment changed from brown to green, with further precipitate formation and discoloration. A brown precipitate was produced when it was reacted with FeCl_3_. These results were identical to standard melanin (Sigma). The identical result for bacterial pigment and standard melanin obtained by chemical characterization primarily confirms the melanin nature of the pigment (Shrishailnath et al. 2010).

The UV absorption spectrum of the melanin produced in this study was analogous to synthetic melanin (Fig 2c), which was reported to be monotonic, broad band and without distinct absorption peaks. It primarily confirmed that the bacterial pigment produced here was melanin (Meredith and Sarna 2006). The FTIR spectra of standard melanin and the produced pigment indicated a high degree of resemblance in the main absorption peaks (Fig. 2d), confirming that purified pigment was melanin (Shrishailnath et al. 2010). The electron paramagnetic resonance (EPR) spectra of standard melanin sigma and bacterial melanin is presented in Fig. 2e and f. The EPR spectrum of bacterial melanin shows *g*-value of 2.009 while *g*-value of standard melanin (Sigma) is 2.004. The EPR analysis of the microbial melanin showed a nearly identical *g*-value compared to standard synthetic melanin (Sigma), and showed resemblance, which confirmed that the purified pigment was melanin (Shrishailnath et al. 2010).

## Conclusion

From the results of this study, it is concluded that the use of this statistical method not only helped in locating the optimum levels of the most significant factors considered with minimum resources and time but also proved to be useful and satisfactory in this process-optimizing exercise. Thus, the optimization of vital nutritional parameters using response surface methodology significantly enhanced the yield of melanin as proved its feasibility for large-scale production by *Brevundimonas* sp. SGJ. So the *Brevundimonas* sp. SGJ can be a potential source for melanin production.

## Electronic supplementary material

Below is the link to the electronic supplementary material.

Fig. S1 **(a)** Phylogenic tree of the *Brevundimonas* sp. SGJ and related organisms **(b)** Melanin producing colonies of *Brevundimonas* sp. SGJ on Nutrient agar plate (**c)** Optimized medium before inocubation (Colorless) and optimized medium after incubation with melanin production (dark brown colored).Fig S2 Pareto chart showing significant effects of factors above the ‘Bonferroni Limit’ and ‘t-value Limit’ and insignificant effect of the factors below the ‘Bonferroni Limit’ and ‘t-value Limit’ X_1_ (pH), X_2_ (temperature), X_3_ (tryptone), X_4_ (yeast extract), X_5_ (beef extract), X_6_ (glucose), X_7_ (l-tyrosine), X_8_ (CuSO_4_), X_9_ (MgSO_4_), X_10_ (K_2_HPO_4_), and X_11_ (NaCl). (DOC 529 kb)
