# Supplementary material for: Optimization of melanin production by Brevundimonas sp. SGJ using response surface methodology
Source: 3 Biotech. 2012 Aug 7;3(3):187–94. doi: 10.1007/s13205-012-0082-4 (PMC3646113; doi:10.1007/s13205-012-0082-4)
Supplement: Supplementary file 1 — Fig. S1 (a) Phylogenic tree of the Brevundimonas sp. SGJ and related organisms (b) Melanin producing colonies of Brevundimonas sp. SGJ on Nutrient agar plate (c) Optimized medium before inocubation (Colorless) and optimized medium after incubation with melanin production (dark brown colored).Fig S2 Pareto chart showing significant effects of factors above the ‘Bonferroni Limit’ and ‘t-value Limit’ and insignificant effect of the factors below the ‘Bonferroni Limit’ and ‘t-value Limit’ X1 (pH), X2 (temperature), X3 (tryptone), X4 (yeast extract), X5 (beef extract), X6 (glucose), X7 (l-tyrosine), X8 (CuSO4), X9 (MgSO4), X10 (K2HPO4), and X11 (NaCl). (DOC 529 kb) [file 13205_2012_82_MOESM1_ESM.doc]

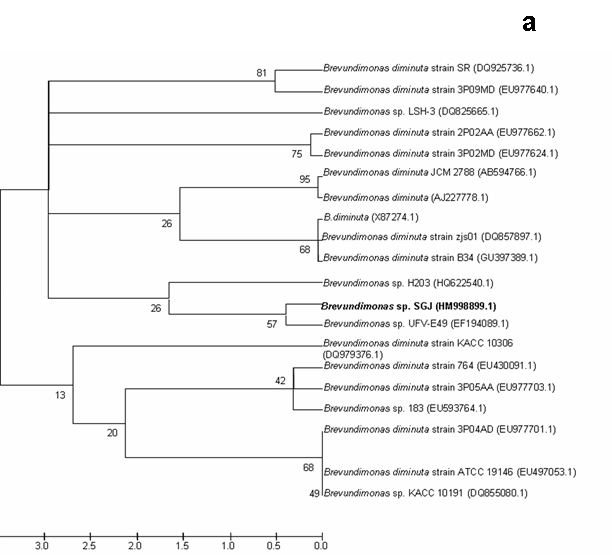


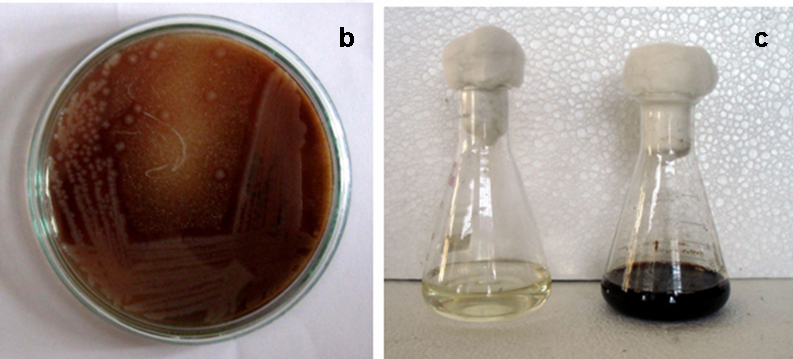


Fig. S1

**
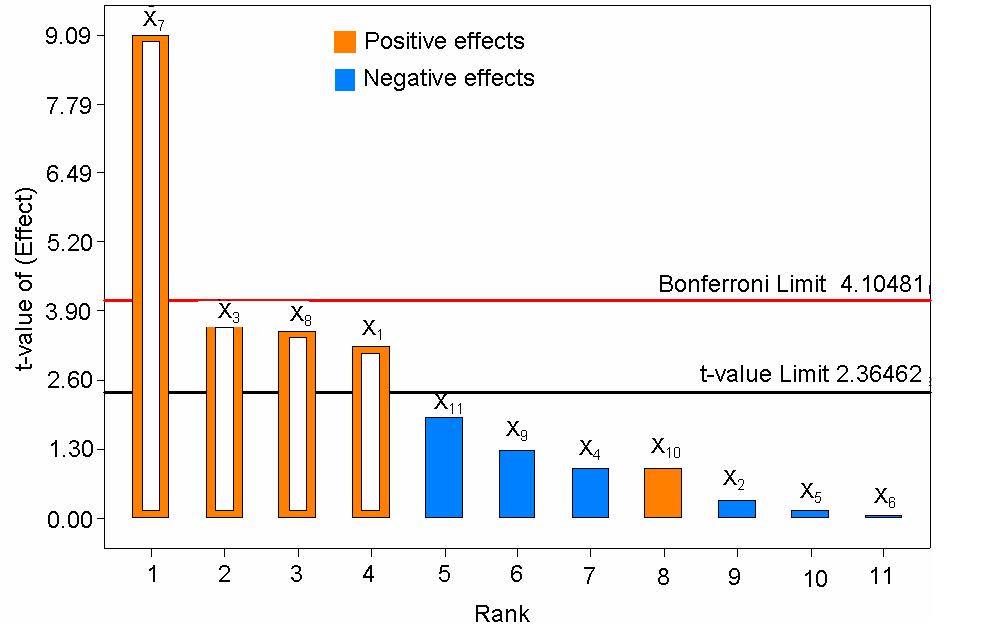
**

Fig. S2

| Run No. | X_1_ | X_2_ | X_3_ | X_4_ | X_5_ | X_6_ | X_7_ | X_8_ | X_9_ | X_10_ | X_11_ | Observed (g l^-1^) | Predicted  (g l^-1^) |
| --- | --- | --- | --- | --- | --- | --- | --- | --- | --- | --- | --- | --- | --- |
| 1 | 1 | 1 | -1 | 1 | 1 | 1 | -1 | -1 | -1 | 1 | -1 | 0.249 | 0.230 |
| 2 | -1 | 1 | 1 | -1 | 1 | 1 | 1 | -1 | -1 | -1 | 1 | 0.357 | 0.365 |
| 3 | 1 | -1 | 1 | 1 | -1 | 1 | 1 | 1 | -1 | -1 | -1 | 0.472 | 0.459 |
| 4 | -1 | 1 | -1 | 1 | 1 | -1 | 1 | 1 | 1 | -1 | -1 | 0.351 | 0.362 |
| 5 | -1 | -1 | 1 | -1 | 1 | 1 | -1 | 1 | 1 | 1 | -1 | 0.305 | 0.286 |
| 6 | -1 | -1 | -1 | 1 | -1 | 1 | 1 | -1 | 1 | 1 | 1 | 0.294 | 0.313 |
| 7 | 1 | -1 | -1 | -1 | 1 | -1 | 1 | 1 | -1 | 1 | 1 | 0.418 | 0.407 |
| 8 | 1 | 1 | -1 | -1 | -1 | 1 | -1 | 1 | 1 | -1 | 1 | 0.256 | 0.279 |
| 9 | 1 | 1 | 1 | -1 | -1 | -1 | 1 | -1 | 1 | 1 | -1 | 0.427 | 0.410 |
| 10 | -1 | 1 | 1 | 1 | -1 | -1 | -1 | 1 | -1 | 1 | 1 | 0.281 | 0.286 |
| 11 | 1 | -1 | 1 | 1 | 1 | -1 | -1 | -1 | 1 | -1 | 1 | 0.249 | 0.282 |
| 12 | -1 | -1 | -1 | -1 | -1 | -1 | -1 | -1 | -1 | -1 | -1 | 0.211 | 0.185 |
| 13 | 0 | 0 | 0 | 0 | 0 | 0 | 0 | 0 | 0 | 0 | 0 | 0.618 | 0.618 |

**Table S1** Design matrix with Observed and predicted response for the experiments performed using Plackett–Burman design for melanin production

-1 low level, +1 high level, 0 center point X_1_ (pH), X_2_ (temperature), X_3_ (tryptone), X_4_ (yeast extract), X_5_ (beef extract), X_6_ (glucose), X_7_ (L-tyrosine), X_8_ (CuSO_4_), X_9_ (MgSO_4_), X_10_ (K_2_HPO_4_), and X_11_ (NaCl)

**Table S2** Statistical analysis of the model by Plackett–Burman design for melanin production

| Source | Sum of Squares | df | Mean Square | F Value | p-value Prob > F |
| --- | --- | --- | --- | --- | --- |
| Model | 0.070731 | 4 | 0.017683 | 29.73781 | 0.0002* |
| X_1_-pH | 0.006165 | 1 | 0.006165 | 10.36854 | 0.0147* |
| X_3_-Tryptone | 0.008112 | 1 | 0.008112 | 13.64235 | 0.0077* |
| X_7_-L-tyrosine | 0.049152 | 1 | 0.049152 | 82.66133 | < 0.0001* |
| X_8_-CuSO_4_ | 0.007301 | 1 | 0.007301 | 12.27901 | 0.0099* |
| Residual | 0.080603 | 1 | 0.080603 |  |  |
| Cor Total | 0.004162 | 7 |  |  |  |

*Significant P values P=<0.05

**Table S3** Analysis of variance (ANOVA) for the fitted quadratic polynomial model of melanin production

| **Source** | **Sum of Squares** | **df** | **Mean Square** | **F Value** | **p-value Prob > F** |
| --- | --- | --- | --- | --- | --- |
| Model | 2.719765 | 14 | 0.194269 | 29.03249 | < 0.0001* |
| X_1_-pH | 0.20856 | 1 | 0.20856 | 31.16827 | < 0.0001* |
| X_3_-Tryptone | 0.0835 | 1 | 0.0835 | 12.47866 | 0.0033* |
| X_7_-L-tyrosine | 0.383419 | 1 | 0.383419 | 57.29995 | < 0.0001* |
| X_8_-CuSO_4_ | 0.152776 | 1 | 0.152776 | 22.83163 | 0.0003* |
| X_1_ X_3_ | 0.06275 | 1 | 0.06275 | 9.377701 | 0.0084* |
| X_1_ X_7_ | 0.128522 | 1 | 0.128522 | 19.20699 | 0.0006* |
| X_1_ X_8_ | 0.047961 | 1 | 0.047961 | 7.167524 | 0.0180* |
| X_3_ X_7_ | 0.018496 | 1 | 0.018496 | 2.764132 | 0.1186 |
| X_3_ X_8_ | 0.025921 | 1 | 0.025921 | 3.87376 | 0.0692 |
| X_7_ X_8_ | 0.095481 | 1 | 0.095481 | 14.26914 | 0.0020* |
| X_1_^2^ | 0.335421 | 1 | 0.335421 | 50.12696 | < 0.0001* |
| X_3_^2^ | 0.130379 | 1 | 0.130379 | 19.48452 | 0.0006* |
| X_7_^2^ | 0.987986 | 1 | 0.987986 | 147.6495 | < 0.0001* |
| X_8_^2^ | 0.698481 | 1 | 0.698481 | 104.3843 | < 0.0001* |
| Residual | 0.09368 | 14 | 0.006691 |  |  |
| Lack of Fit | 0.084955 | 10 | 0.008496 | 3.894886 | 0.1010 |
| Pure Error | 0.008725 | 4 | 0.002181 | 29.03249 | < 0.0001* |
| Cor Total | 2.813445 | 28 | 0.194269 | 31.16827 | < 0.0001* |

*Significant P values P=<0.05

**Table S4** The Box-Behnken design matrix for coded variables along with actual and predicted responses for melanin production

| Standard  order | Factor X_1_  (pH) | Factor  X_3_  (Tryptone) | Factor  X_7_  (L-tyrosine) | Factor X_8_  (CuSO_4_) | Actual response (Y g l^-1^) | Predicated response (Y_1_ g l^-1^) |
| --- | --- | --- | --- | --- | --- | --- |
| 1 | -1 | -1 | 0 | 0 | 0.993 | 0.956 |
| 2 | 1 | -1 | 0 | 0 | 0.431 | 0.442 |
| 3 | -1 | 1 | 0 | 0 | 0.965 | 0.872 |
| 4 | 1 | 1 | 0 | 0 | 0.904 | 0.859 |
| 5 | 0 | 0 | -1 | -1 | 0.337 | 0.296 |
| 6 | 0 | 0 | 1 | -1 | 0.321 | 0.344 |
| 7 | 0 | 0 | -1 | 1 | 0.318 | 0.212 |
| 8 | 0 | 0 | 1 | 1 | 0.92 | 0.879 |
| 9 | -1 | 0 | 0 | -1 | 0.496 | 0.505 |
| 10 | 1 | 0 | 0 | -1 | 0.458 | 0.461 |
| 11 | -1 | 0 | 0 | 1 | 0.862 | 0.950 |
| 12 | 1 | 0 | 0 | 1 | 0.386 | 0.467 |
| 13 | 0 | -1 | -1 | 0 | 0.292 | 0.425 |
| 14 | 0 | 1 | -1 | 0 | 0.383 | 0.456 |
| 15 | 0 | -1 | 1 | 0 | 0.629 | 0.647 |
| 16 | 0 | 1 | 1 | 0 | 0.992 | 0.949 |
| 17 | -1 | 0 | -1 | 0 | 0.318 | 0.307 |
| 18 | 1 | 0 | -1 | 0 | 0.454 | 0.402 |
| 19 | -1 | 0 | 1 | 0 | 0.983 | 1.023 |
| 20 | 1 | 0 | 1 | 0 | 0.402 | 0.401 |
| 21 | 0 | -1 | 0 | -1 | 0.461 | 0.405 |
| 22 | 0 | 1 | 0 | -1 | 0.673 | 0.732 |
| 23 | 0 | -1 | 0 | 1 | 0.862 | 0.791 |
| 24 | 0 | 1 | 0 | 1 | 0.752 | 0.797 |
| 25 | 0 | 0 | 0 | 0 | 1.156 | 1.151 |
| 26 | 0 | 0 | 0 | 0 | 1.194 | 1.151 |
| 27 | 0 | 0 | 0 | 0 | 1.072 | 1.151 |
| 28 | 0 | 0 | 0 | 0 | 1.169 | 1.151 |
| 29 | 0 | 0 | 0 | 0 | 1.168 | 1.151 |
